# Evolution of selfing syndrome and its influence on genetic diversity and inbreeding: A range‐wide study in *Oenothera primiveris*

**DOI:** 10.1002/ajb2.1861

**Published:** 2022-05-21

**Authors:** Anita Cisternas‐Fuentes, Tania Jogesh, Geoffrey T. Broadhead, Robert A. Raguso, Krissa A. Skogen, Jeremie B. Fant

**Affiliations:** ^1^ Negaunee Institute for Plant Conservation Science and Action Chicago Botanic Garden 1000 Lake Cook Road Glencoe Illinois 60035 USA; ^2^ Plant Biology and Conservation Northwestern University 2205 Tech Drive Evanston Illinois 60208 USA; ^3^ Department of Biological Science Clemson University 132 Long Hall Clemson South Carolina 29631 USA; ^4^ Department of Entomology and Nematology University of Florida 1881 Natural Area Drive Gainesville Florida 32611 USA; ^5^ Department of Neurobiology and Behavior Cornell University W361 Mudd Hall Ithaca New York 14853 USA

**Keywords:** breeding system, genetic diversity, inbreeding, mating system, Onagraceae, RADseq, self‐incompatibility

## Abstract

**Premise:**

To avoid inbreeding depression, plants have evolved diverse breeding systems to favor outcrossing, such as self‐incompatibility. However, changes in biotic and abiotic conditions can result in selective pressures that lead to a breakdown in self‐incompatibility. The shift to increased selfing is commonly associated with reduced floral features, lower attractiveness to pollinators, and increased inbreeding. We tested the hypothesis that the loss of self‐incompatibility, a shift to self‐fertilization (autogamy), and concomitant evolution of the selfing syndrome (reduction in floral traits associated with cross‐fertilization) will lead to increased inbreeding and population differentiation in *Oenothera primiveris*. Across its range, this species exhibits a shift in its breeding system and floral traits from a self‐incompatible population with large flowers to self‐compatible populations with smaller flowers.

**Methods:**

We conducted a breeding system assessment, evaluated floral traits in the field and under controlled conditions, and measured population genetic parameters using RADseq data.

**Results:**

Our results reveal a bimodal transition to the selfing syndrome from the west to the east of the range of *O. primiveris*. This shift includes variation in the breeding system and the mating system, a reduction in floral traits (flower diameter, herkogamy, and scent production), a shift to greater autogamy, reduced genetic diversity, and increased inbreeding.

**Conclusions:**

The observed variation highlights the importance of range‐wide studies to understand breeding system variation and the evolution of the selfing syndrome within populations and species.

The cost of sexual reproduction is a well‐established tenet of evolutionary biology (Maynard‐Smith, 1978; Otto, 2009; Gibson et al., 2017). Yet, for a majority of eukaryotic species, sex and recombination are the predominant form of reproduction (Charlesworth, 1989). Among flowering plants the complete spectrum of sexual expression is found, from separate sexes to parthenogenesis, the most common being hermaphroditism (Charlesworth, 1980), whereby sexual reproduction can occur via selfing and outcrossing. However, despite the prevalence of hermaphroditism, 50% of angiosperm species have also evolved self‐incompatibility (SI) systems, thereby making them obligate outcrossers (Igic et al., 2008). This is intriguing, given that outcrossing is considered evolutionarily expensive (Fisher, 1941; Barrett and Harder, 1996). One cost of outcrossing is that each offspring inherits only one copy of an individual's chromosomes, and recombination can break down important gene associations (Maynard‐Smith, 1986; Otto, 2009), although the relative impact is somewhat dependent on the relatedness between parents (Uyenoyama, 1984). By contrast, self‐fertilization has many benefits, including increasing the proportion of an individual's genes that are passed on (known as transmission advantage; Holsinger, 1991) and providing reproductive assurance (Charlesworth, 1989; Barrett and Harder, 1996). However, the prevalence of outcrossing suggests that the advantages of self‐fertilization might be short‐term and are offset by the increased potential for inbreeding depression, whereby fitness is reduced due to the accumulation and expression of the genetic load (Charlesworth and Willis, 2009; Barrett and Harder, 2017). The avoidance of inbreeding depression is proposed as the main reason flowering plants have evolved such a diversity of breeding and mating systems in spite of the high cost of sexual reproduction (Stebbins, 1974; Barrett, 2002).

The role that inbreeding depression plays in the evolution of sexual reproduction is determined by the genetic load of the population and the rate of inbreeding (Whitlock, 2002). The genetic load that a population carries, and therefore likelihood of expressing inbreeding depression, will depend on many interacting factors, including life‐history traits (Duminil et al., 2009), population size (Angeloni et al., 2011), the number of founders, and population growth rates (Biebach and Keller, 2010). In populations that are large or that experience high gene flow, the genetic load is often hidden in a heterozygous state (Lohr and Haag, 2015), and a large genetic load can be maintained without significant impacts to the average population fitness (Theodorou and Couvet, 2006). However, if populations become small or there is a shift in mating system toward elevated inbreeding, the genetic load can be expressed, resulting in a decline in population fitness (Paland and Schmid, 2003). In these latter situations, purging of deleterious alleles can occur over time, resulting in an increase in fitness and the evolution of a stable state (Crow, 1970; Dudash and Carr, 1998; Crnokrak and Barrett, 2002). Hence, the cost of inbreeding depression during the transition to selfing will depend on the extent of the genetic load and how it is expressed (Goodwillie et al., 2005; Voillemot et al., 2018).

Plant breeding systems (per Neal and Anderson, 2005) shape the mating patterns within a population by determining the extent to which selfing can occur (Charlesworth, 2006; Raduski et al., 2012). Self‐incompatible populations consist of obligate outcrossing plants whose mating system is expected to be predominantly outcrossing (Schoen and Lloyd, 1992). However, for populations that are partially or completely self‐compatible, the mating system can span from outcrossing to mixed mating to completely selfing (Holsinger, 1991; Goodwillie et al., 2005). The loss of self‐incompatibility (SI) has occurred multiple times in angiosperms (Stebbins, 1974; Raven, 1979; Igic et al., 2008). Once self‐compatibility (SC) has evolved, the mating system of the population will depend on the frequency of SC individuals in the population and the rate of selfing (Holsinger, 1991; Whitehead et al., 2018). Evolutionary models predict that the most stable state for a population is either complete selfing or complete outcrossing (Lande and Schemske, 1985), yet the empirical evidence suggests that mixed mating is the most common state in nature (Goodwillie et al., 2005). Conditions that favor the persistence of self‐fertilization include small population size, low density, increased fragmentation and isolation, and limited number of potential mates, pollinators, or pollen (Karron et al., 1995, 2012; Goodwillie et al., 2005; Busch and Schoen, 2008; Eckert et al., 2010; Devaux et al., 2014; Voillemot and Pannell, 2017b; Whitehead et al., 2018). Under these conditions, individuals with leaky SI or SC have a fitness advantage over those that are strictly SI. The conflict between the advantage of self‐fertilization for reproductive assurance in the short term (Charlesworth, 2006) and the disadvantages of increased inbreeding will dictate the evolutionary trajectory of the population, influencing both floral traits and population genetic patterns (Eckert et al., 2010; Wright et al., 2013; Cheptou, 2019).

In situations where self‐fertilization is evolutionarily advantageous, selection will favor changes in traits that further facilitate selfing (Darwin, 1876; Ornduff, 1969; Snell and Aarssen, 2005; Sicard et al., 2011; Shimizu and Tsuchimatsu, 2015), including reductions in flower size (Sicard and Lenhard, 2011; Duncan and Rausher, 2013b; Summers et al., 2015; Tedder et al., 2015); reduced nectar (Sicard and Lenhard, 2011), pollen (Tedder et al., 2015), or scent production (Raguso et al., 2007; Sicard et al., 2011; Doubleday et al., 2013); changes in the intensity of petal colors (Button et al., 2012; Duncan and Rausher, 2013a); and early or increased growth rates (Rifkin et al., 2019). These changes may be the result of abiotic (Evans et al., 2005, 2009) and biotic factors acting individually or in concert. An important change in floral morphology associated with increased self‐fertilization is the reduction in herkogamy, the spatial separation between the anthers and stigma (Webb and Lloyd, 1986; Bodbyl Roels and Kelly, 2011; Opedal, 2018; Cheptou, 2019). The close proximity of anthers and stigma in SC individuals can increase autogamy (self‐pollination within a flower) and thereby provide reproductive assurance under suboptimal environmental conditions (Charlesworth, 2006; Cheptou, 2019) or when pollen is limited (Fishman and Willis, 2008; Bodbyl Roels and Kelly, 2011; Toräng et al., 2017). These changes can have consequences for genetic parameters, including elevated rates of inbreeding, reduction in genetic diversity, increased genetic differentiation among populations (Barrett and Harder, 1996), and ultimately the expression of the genetic load (Charlesworth and Willis, 2009; Barrett and Harder, 2017).

Whether selfing lineages persist depends on the interplay of selective advantages of reproductive assurance and the fitness cost of reproduction and survival from the expression of the genetic load. If the rate of inbreeding increases too rapidly, fitness declines and genetic diversity that is lost through selective sweeps prior to purging can impact population viability and long‐term persistence, and therefore the extent to which the population can recover over the long term (Frankham, 2005). Hence, it has been postulated that the conditions that would favor a shift to SC include slow changes in rates of inbreeding over many generations and large effective population size to minimize loss of diversity from drift (López‐Cortegano et al., 2016; Caballero et al., 2017). Under this scenario, the potential negative impacts of the genetic load are reduced and potentially purged, minimizing the cost of inbreeding and facilitating the transition to the evolution of the selfing syndrome.

To date, most studies investigating the evolution of the selfing syndrome have focused on comparisons between closely related species and taxa that differ in key characters of interest (Charlesworth, 2006). Intraspecific studies that compare populations with different rates of selfing can provide valuable insight into the early stages of reproductive isolation and divergence, important for speciation (Cutter, 2019). Previous intraspecific studies looking at the evolution of the selfing syndrome have demonstrated some of the predicted changes, but none have found a complete transition to the selfing syndrome (Button et al., 2012; Summers et al., 2015; Voillemot and Pannell, 2017b). In a recent study of *Linaria cavanillesii* Chav., a shift to SC was associated with increased autogamy, reduced inbreeding depression, and differentiation from a SI population, but the authors found no evidence of changes in floral morphology or mating system (Voillemot and Pannell, 2017a, 2017b), suggesting that this taxon was in the early stages of evolution of the selfing syndrome (Voillemot et al., 2018). Conversely, in *Camissoniopsis cheiranthifolia* (Hornem. ex Spreng.) W.L. Wagner & Hoch, a shift in floral morphology, gradual shift in the mating system from outcrossing to selfing, and an increase in autogamy were not associated with significant increases in the inbreeding coefficient or evidence for loss of inbreeding depression, suggesting that mixed mating is maintained (Button et al., 2012; Dart et al., 2012; Dart and Eckert, 2013). However, these results may be driven by hybridization and taxonomic uncertainty in this system (López‐Villalobos and Eckert, 2019). Additional intraspecific studies (sampling range‐wide and including multiple populations) are needed to better understand how the transition from SI to SC and associated changes in mating system, floral traits, and genetic diversity drive the evolution of the selfing syndrome (Foxe et al., 2010).

In this study, we investigate the loss of self‐incompatibility (SI) and the transition from mainly outcrossing to increased selfing, leading to the evolution of the selfing syndrome in the desert evening primrose, *Oenothera primiveris* A. Gray (Onagraceae). This species exhibits a population‐level, longitudinal shift in variation in floral traits and breeding system suggestive of an evolutionary transition from SI to SC and an increase in selfing. Specifically, large SI flowers occur in the western portion of the range, whereas eastern populations have reduced, SC flowers (Wagner, 2005). It is known that SI systems in other members of the family are variable and that leaky SI has evolved in some taxa; but in the genus *Oenothera*, nearly all species are SI, except for seven that show some level of SC (Klein, 1964; Steiner and Stubbe, 1984; Wagner, 2005; Theiss et al., 2010). The species *O. primiveris* provides a favorable system to investigate the stepwise evolution of the selfing syndrome within a species, as well as the importance of genetic changes associated with shifts in breeding and mating systems. We test the hypothesis that the shift toward increased selfing is associated with changes in floral traits, higher rates of autogamy, increased inbreeding, lower genetic diversity, and greater population differentiation. We sampled eight populations across the distribution of *O. primiveris* and collected data on floral traits, pollinator visitation, and genetic parameters and conducted a breeding system assessment on plants grown under controlled conditions.

## MATERIALS AND METHODS

### Study system

*Oenothera primiveris*, the only member of *Oenothera* sect. *Eremia*, is an herbaceous annual or short‐lived perennial that occurs in sand dune habitats in the Mojave, Sonoran, and Chihuahuan Deserts of the United States and Mexico (Wagner, 2005) and in patchy populations in sandy/rocky soils of dry washes (ephemeral streams/river systems). Populations that occur in dune systems are large, likely due to the greater availability of suitable habitat compared with those in the center and east of the distribution, which occur in sandy/gravel washes that are more limited in size and therefore have smaller population sizes. Fruits and seeds are likely dispersed along washes during rain events. Floral traits vary across the distribution, but all flowers are pale yellow with evening anthesis, remaining open until the next morning when they senesce. Populations in the western and central portions of the range exhibit floral traits associated with long‐distance pollination by hawkmoths (large, fragrant flowers that produce nectar) while those in the eastern portion of the range have flowers with reduced floral characters (small flowers and anthers surrounding the stigma at anthesis), commonly associated with a transition to predominantly selfing, and are presumed to have little or no cross‐pollination (Wagner, 2005).

**Figure 1:**
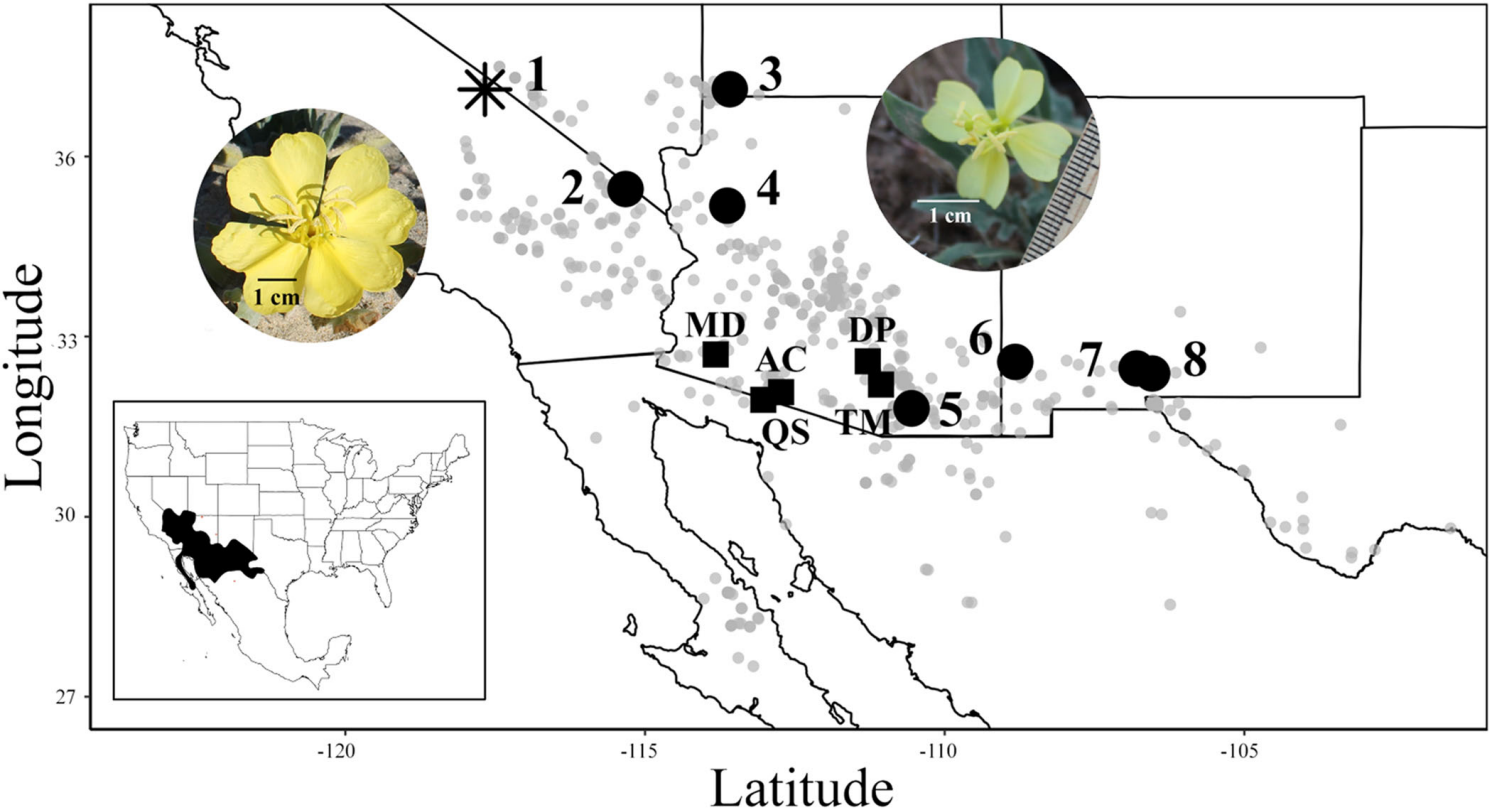
Distribution of *Oenothera primiveris* in western USA and northern Mexico. Gray circles represent herbarium records for the species (Global Biodiversity Information Facility, 2020). Sampled populations (Table 1) are denoted by black circles and an asterisk, the latter of which is the only population described as fully self‐incompatible by Wagner (2005). Populations sampled in 2001 are denoted by black squares (see methods, Appendix S8). Pictures of *O. primiveris* show the variation in flower size observed across the distribution, with large flowers in the west and small flowers in the east.

Eight populations of *O. primiveris* spanning the geographic range of the species in the United States were studied, representing the expected spectrum of breeding system and variation in flower size (Figure 1; Table 1). Of these, four populations were located in the west of the distribution, three with large flowers (Pop 1: Eureka Dunes, the only known population to exhibit complete SI [Wagner, 2005]; Pop 2: Nipton Road; Pop 3: T‐Bone Hill) and one with small flowers (Pop 4: Hackberry Road). In addition, four focal populations were located in the east of the distribution, all of which had small flowers (Pop 5: Whetstone Mountains; Pop 6: Dona Ana; Pop 7: White Box Canyon; and Pop 8: Aguirre Springs). Census population size was estimated in six of the populations and was classified as large (>250 plants; Pops 3 and 4), medium (~100–250 plants; Pops 1 and 2), or small (<100 plants; Pops 6 and 7) (Table 2). Voucher specimens from each population were deposited at the Nancy Rich Poole Herbarium (Chicago Botanic Garden, CHIC) and the United States National Herbarium at the Smithsonian Institution (US). Data collected for each population are summarized below and in Table 1.

**Table 1:** *Oenothera primiveris* populations included in the study and summary of floral trait data collected in the field (F) or in the growth‐chamber experiment (G): scent (S), morphology (M), and pollinator observations (PO), population genetic parameters (PG), and breeding system assessments (BS). Mean flower diameter and herkogamy are reported in millimeters, and the standard error is provided in parentheses. Average self‐compatibility index (SCI) is the ratio of seeds produced by self‐pollination compared to outcross pollination; “breeding system” records the types of breeding system observed in the population: SC means that all individuals were self‐compatible, and SI/SC means that both types of breeding system were observed)

Population	Location	Latitude, longitude	Collector number	Data collected	Diameter	Herkogamy	SCI	Breeding system
Pop 1	Eureka Dunes, CA	37.1189,−117.672	LOL 590	F: S, M, POG: PG, BS, M	F: 53.83 (1.3) G: 57.47 (1.36)	F: 7.98 (0.63) G: 7.6 (0.6)	0.13	SI/SC
Pop 2	Nipton Rd, NV	35.463,−115.319	LOL 183	F: S, M, POG: PG, BS, M	F: 60.25 (1.58) G: 59.58 (1.8)	F: 10.53 (0.51) G: 5.51 (0.57)	0.39	SI/SC
Pop 3	T‐Bone Hill, UT	37.133,−113.582	LOL 201	F: S, M, POG: PG, BS, M	F: 71.45 (2.05) G: 59.88 (3.27)	F: 10.91 (0.94) G: 9.04 (2.06)	0.29	SI/SC
Pop 4	Hackberry Rd, AZ	35.186,−113.627	LOL 240	F: S, M, POG: PG, BS, M	F: 31.33 (0.83) G: 30.47 (1.02)	F: 2.07 (0.65) G:0.6 (0.37)	0.67	SI/SC
Pop 5	Whetstone Mt, AZ	31.814,−110.551	LOL 584	F: S, M	F: 36.02 (2.05)	F: 1.19 (1.32)	NA	NA
Pop 6	Dona Ana, NM	32.472,−106.799	LOL 579	G: PG, BS, M	G: 38.47 (1.23)	G: 0.85 (0.49)	0.69	SC
Pop 7	White Box Canyon, NM	32.587,−108.817	LOL 583	G: PG, BS, M	G: 38.84 (1.41)	G: − 0.31 (0.51)	0.81	SC
Pop 8	Aguirre Springs, NM	32.391,−106.535	LOL 604	G: BS, M	G: 34.38 (1.12)	G: − 0.28 (0.3)	0.77	SI/SC

**Table 2:** Genetic diversity parameters by population of *Oenothera primiveris.*

Population	Population size	Sample size	%P	*N*	*N*_A_	*H*_O_	*H*_E_	*F*_IS_	*N*_E_ (CI 95%)	Selfing rate (SE)
Pop 1	Medium	20	61.7	1.62	1.21	0.13	0.13	0.03	47.4 (39–60)	0.17 (0.04)
Pop 2	Medium	20	45.9	1.46	1.18	0.09	0.11	0.13	32.3 (27–40)	0.32 (0.04)
Pop 3	Large	20	54.2	1.54	1.19	0.10	0.12	0.12	28.2 (24–34)	0.31 (0.05)
Pop 4	Large	20	21.5	1.22	1.11	0.08	0.06	−0.15	11.8 (9–16)	0.2 (0.05)
Pop 6	Small	19	16.6	1.17	1.08	0.02	0.05	0.58	3.8 (3–6)	0.57 (0.12)
Pop 7	Small	20	18.3	1.18	1.08	0.02	0.05	0.55	16.1 (12 22)	0.6 (0.05)

*Notes*: %P = percentage of polymorphic SNP, *N* = mean number of alleles per locus, *N*_A_ = number of effective alleles, *H*_O_ = observed heterozygosity, *H*_E_ = expected heterozygosity, *F*_IS_ = Inbreeding coefficient, and *N*_E_ = estimated effective population size and confidence interval (CI).

### Breeding system assessment

Breeding system assessments were conducted under controlled conditions in growth chambers (CMP6050; Conviron, Winnipeg, Manitoba, Canada) at the Chicago Botanic Garden (Glencoe, Illinois, USA) for seven of the eight populations visited. Plants were grown from field‐collected fruits that were dried to 20% relative humidity before cleaning. Multiple fruits from each maternal line were collected in each population; seeds from each maternal line were cleaned and pooled. Cleaned seeds were maintained at 4°C before germination. Seeds were surface sterilized by submerging for 5 min in a 20% bleach solution and rinsed in deionized water before being placed on Petri dishes with 1.5% agar with 1 mL of Plant Preservative Mixture (Plant Cell Technology, Washington, D.C., USA; Guri and Patel, 1998). Plates were then placed in cold stratification at 4°C for 7 d, followed by 5–7 d in an incubator with diurnal cyclic conditions of 25°C for 12 h (day) and 15°C for 12 h (night). Seeds were nicked with forceps to facilitate higher germination success and were then surface sterilized before placing them in fresh agar plates. Plates were then returned to the incubator under the conditions described above until germination. Seedlings were planted in a germination potting mix and watered every 3 d for the first 3 wk of establishment and then twice a week thereafter. When rosettes reached ~3 cm in diameter, they were transplanted into larger (6.4 cm^2^) pots using a 3:1 ratio of regular potting soil and perlite. We aimed for 15 maternal lines for each of the seven populations for which we had seeds, in order to evaluate breeding system, autogamy, and morphological traits, although the limited numbers of fruits collected from populations 6 and 7, poor germination, and limited number of flowers produced by all populations reduced the final number of individuals and maternal lines used for each experiment (for sample size information, see Appendices S1 and S2).

The breeding system of each population was assessed via controlled crosses (treatments: within population outcross and self) on newly opened flowers to ensure maximum pollen viability and stigma receptivity. Flowers used as pollen recipients for both treatments were emasculated (anthers removed) with forceps prior to hand‐pollination to reduce cross‐contamination. Anthers were removed from pollen donors using forceps, and then all anthers were used to saturate the clean stigmatic surface of the recipient flower with pollen. In the case of within‐population outcrosses, anthers were placed in clean Petri dishes before performing the controlled cross, while in the self‐pollinations, the anthers were removed and rubbed onto the stigma of the same flower immediately. Forceps were cleaned with a 70% ethanol solution between crosses to prevent unintentional pollen transfer. A subset of plants was also used to assess seed set via autogamy, because variation in mating system and reductions in floral traits may contribute to variation in autogamous self‐pollination among populations. Jeweler's tags were used to record maternal and paternal information and cross‐type, and once flowers had senesced, a colored wire was tied around the top of the capsule to prevent the release of seeds as the fruit matured. Fruits were collected when mature, and seed number was recorded. Crosses were considered incompatible if no seeds were produced.

To assess SI at the population level, an average self‐compatibility index (SCI) was calculated for each maternal line included in the study and then for each population (Appendix S1). This index represents the variation in self‐compatibility across maternal lines of a single population. SCI ranges from 0 to 1, where 0 represents full SI and 1 represents fully SC. SCI was calculated as the ratio of seeds produced by self‐pollination to those produced from within‐population crosses for each maternal line in a population (Zapata and Arroyo, 1978; Ruhsam et al., 2010). An average was calculated when multiple fruits per cross were available within a maternal line. The ratio between self and outcross seeds was calculated for each maternal line and then averaged across all maternal lines in each population to obtain a population‐level estimate.

### Floral traits

Floral trait data were collected to determine whether the changes associated with the selfing syndrome had evolved in populations showing SC and higher autogamy. Floral traits were measured in the field in March and April 2015 and March 2016 (Table 1) in the four western populations (Pops 1, 2, 3, and 4) and one of the eastern populations (Pop 5), the latter of which had a small census size, limiting data and sample collection. Floral morphology was collected from 9–30 individuals/population. Flowers were excised from the plants at the base of the ovary, and the following morphological traits were measured (to the nearest 0.01 mm using digital calipers) on one flower per plant following Jogesh et al. (2016): corolla diameter, floral tube length, floral flare, and herkogamy (stigma‐anther separation). Floral morphology was also measured on a subset of plants grown under controlled conditions (Breeding System Assessment, above) and included the same traits with the exception of floral flare, which was not measured, to avoid damaging floral tissues and cross‐contamination of pollen, given that the same flowers were used in the breeding system treatments. Herkogamy was calculated from separate measures of style and filament lengths. Both measurements were taken from the base to the apex of the stigma or the anthers, and the filament measurement was subtracted from that of the style to calculate herkogamy. Field and growth‐chamber measurements of morphology were compared to determine the extent to which differences observed in the field are maintained under common growing conditions.

Floral scent was collected in situ on 9–30 individuals/population from five populations (Table 1) using dynamic headspace collection methods (Raguso and Pellmyr, 1998; Galen et al., 2011) at floral anthesis. Measurements of floral morphology and floral scent collected in the field in 2015 and 2016 were from the same individual flowers. We sampled one flower per plant and collected floral scent immediately after anthesis, between 1800 and 2000 hours. Each flower was enclosed within a nylon resin (Reynolds America, Winston‐Salem, North Carolina, USA) oven bag (12 × 15 cm, 270 mL vol.), which was cinched around the floral tube (hypanthium) with plastic ties. Floral volatiles were collected in a cartridge constructed from a glass Pasteur pipette, containing an adsorbent material (10 mg of 80–100 mesh Super Q; Alltech Associates, Waukegan, Illinois, USA) held in place using silanized quartz wool (Supelco, Berwick, Pennsylvania, USA). Air from the floral headspace, concentrated in the enclosing bag, was pulled through the cartridge at a flow rate of 200 mL/min using a 9 V battery–operated personal air sampler vacuum pump (Supelco). After 60 min of sampling, the cartridges were removed, and volatiles were eluted with 200 μL of GC‐MS quality hexane (Merck, Kenilworth, New Jersey, USA) into Teflon‐capped borosilicate glass vials. Samples were stored on ice while in the field and then at −20°C until they were processed, to avoid evaporation. Before analysis, we concentrated the samples to a uniform volume of 50 μL using gaseous N_2_ and added 5 μL of 0.03% toluene in hexane (= 23 ng) as an internal standard. One µL aliquot of each sample was injected into a Shimadzu GC‐17A gas chromatograph equipped with a Shimadzu QP5000 quadrupole, electron ionization (EI) mass spectrometer (Shimadzu Scientific Instruments, Columbia, Maryland, USA) as a detector. All analyses were made using splitless injections on a polar GC column (diameter 0.25 mm, length 30 m, film thickness 0.25 µm (Econo Cap's carbowax coating, known as EC WAX; Alltech Associates, Deerfield, Illinois, USA), using ultra‐high‐purity (99.999%) helium as a mobile phase (split ratio 12:1, a constant flow of 1 mL/min). The GC temperature and pressure parameters (injection port temp. 240°C, detector temp. 260°C, initial temp. 40°C, hold time 2 min, increased at 15°C/min to 260°C, hold time 2.38 min) were optimized to resolve floral volatiles common to *Oenothera* species with a total run time of 19 min/sample, allowing us to efficiently process high sample replicates. EI mass spectra (70 eV) were collected from m/z 40–350 (daltons) at a detector voltage of 70 eV, with a scan speed of 1000 and a scan interval of 0.29 s. Compounds were tentatively identified using computerized mass spectral libraries (Wiley Registry of Mass Spectral Data, National Institute of Standards and Technology) and Adams (>120,000 mass spectra; Adams, 2007) and were verified whenever possible by comparing mass spectra and standardized retention indices with those of authentic standards. Peak areas were integrated using Shimadzu's GCMSolutions software, and were normalized for slight differences in final sample volume using the internal standard. Emission rates were calculated using the internal standard and were expressed as ng toluene equivalents per flower per h. Floral scent was also collected from greenhouse‐grown plants at the University of Arizona in 2001 using a modified but comparable protocol (see Raguso et al., 2007) and were compared with the field‐collected data collected here, to assess the extent to which overall scent compounds and emission rates are similar to those observed when plants are grown in a controlled setting.

### Next‐generation sequencing and estimating genetic parameters

Total genomic DNA was extracted from leaf tissue collected in the field following a modified cetyltrimethylammonium (CTAB) protocol developed by Doyle and Doyle (1987). Restriction site‐associated DNA sequencing (RADseq) was used to identify single‐nucleotide polymorphisms (SNPs) across six populations of *O. primiveris* (Pops 1, 2, 3, 4, 6, and 7; Table 2). We sequenced 20 individuals for each population, except Pop 6, where only 19 samples were collected. RADseq allows for a cost‐effective random representation of the genome when there is no previous sequence information available (Davey and Blaxter, 2010). The samples were prepared using the RADseq method developed by Elshire et al. (2011). Two genomic libraries of 96 unique barcodes were constructed through the digestion, ligation, and polymerase chain reaction (PCR) of each sample. To avoid any batch effect, each genomic library contained half of the samples from each population and included samples of a species run previously with this technique as a positive control (*O. harringtonii* W.L. Wagner, Stockh & W.M. Klein). For the digestion, the genome was cut with ApeKI (no. R0643; New England Biolabs, Ipswich, Massachusetts, USA) and then ligated with oligonucleotides, which included specific Illumina primers and a unique barcode. Unique 96 barcodes were obtained according to the specification of the protocol from Integrated DNA Technology (IDT, Coralville, Iowa, USA). These barcodes ensured that only fragments containing the specific primers were amplified. Each PCR was carried out independently for all samples, and each library was then quantified using High sensitivity QubitTM kit (dsDNA HS Assay Kit; Thermo Fisher Scientific, Waltham, Massachusetts, USA) and then pooled in the final step before sequencing to ensure that an equivalent amount of each sample was present in the final genomic library. Sequencing was performed using llumina HiSeq Sequencing (150 bp paired‐end reads) at the Center for Genetic Medicine at Northwestern University.

To build loci and detect haplotypes for each individual, we used the denovo.map.pl pipeline of Stacks version 1.28 (Catchen et al., 2013). Different combinations of filtering parameter used in Ustacks and Cstacks were tested following the recommendation by Paris et al. (2017). The parameter combination that provided the highest and more conserved number of alleles included minimum depth of coverage to create a stack (*m* = 3), maximum distance to create stacks (*M* = 3), and number of mismatches allowed while creating the catalog (*n* = 2). We used three individuals with the highest number of reads from each population to build the catalog. Finally, we used populations to identify SNPs, restricting the data to only the first locus per read. Only SNPs that were present in 70% of the individuals were considered in the final data set. Genepop and Structure outputs were obtained from Stacks and used for population genetic analysis.

### Statistical analysis

A one‐way ANOVA was used to assess population‐level differences in floral traits from measurements collected in the field: flower diameter (calculated as the mean of the two measurements), floral flare (field only), herkogamy, floral tube length, and total scent emission rates. Differences in overall scent composition and morphology between populations were visualized using nonmetric multidimensional scaling (NMDS) of Bray‐Curtis distance metrics, and statistical differences were determined using nonparametric analysis of similarities (ANOSIM; Clarke, 1993). We used a linear mixed model to analyze floral traits measured in the greenhouse (floral diameter, herkogamy, and floral tube length), with maternal line as a random effect, using the R package nlme (Pinheiro et al., 2022). The average number of seeds produced through autogamy was calculated for each maternal line in the population, using an average of seeds produced by the maternal line. We evaluated differences across populations in SCI and average number of autogamous seeds produced using a generalized linear model with a quasibinomial (for SCI) or quasipoisson distribution (for autogamous seeds). Mean values of autogamous seeds produced for each population were correlated with mean flower diameter and herkogamy for each population (values used to perform the analysis can be found in Appendix S2). Tukey post hoc tests were conducted to determine which populations differed in floral traits, SCI, and autogamous seed number. We also calculated pollinator visitation rates (number of visits per flower per hour) per population, but visits were not frequent enough for statistical analysis (Appendix S3).

The following population genetic parameters were estimated in GenAlEx (Peakall and Smouse, 2012): percentage of polymorphic loci (%P), mean number of alleles per locus (*N*), number of effective alleles (*N*_A_), observed and expected heterozygosity (*H*_O_ and *H*_E_), and differentiation index between populations (*F*_ST_). Inbreeding coefficients (*F*_IS_) were obtained through Genepop (Rousset, 2008), selfing rates estimates were estimated through SPAGEDI (Hardy and Vekemans, 2002; David et al., 2007; Hardy, 2016), and estimations of effective population size (*N*_E_) were obtained in NeEstimator version 2 (Do et al., 2014) using the linkage‐disequilibrium method.

To visualize population differentiation between populations, we used a Bayesian clustering analysis in STRUCTURE (Pritchard et al., 2000). We ran simulations using a model that infers population structure with admixture from one to eight clusters with 100,000 MCMC iterations followed by 100,000,000 burn‐in chains for 20 independent replicates. To identify the most likely number of *K* clusters, delta *K* was calculated as described in Evanno et al. (2005).

To test the role of breeding system and flower size on genetic diversity, we used the mean SCI, mean flower diameter, and herkogamy as explanatory variables to evaluate differences between genetic parameters (%P, N, *N*_A_, *H*_O_, *H*_E_, and *F*_IS_; Table 2), the mean number of autogamous seeds produced (Appendix S2), and the effective population size, *N*_E_ (Table 2). All analyses were performed in R version 3.3.3 (R Core Team, 2017).

## RESULTS

### Breeding system assessment

**Figure 2:**
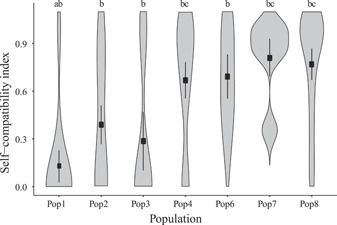
Violin plots of the variation in the self‐compatibility index in seven populations of *Oenothera primiveris* reveal limited SC in Pop 1, moderate frequency of SC in Pops 2 and 3, and high levels of SC in Pops 4 and 6–8. Pop 1 had a significantly lower SCI than three of the other populations, which were not different from each other. Populations sharing a letter are not significantly different from each other (*P* > 0.05).

The SCI values revealed that both SI and SC individuals were found in five of the seven populations evaluated, with the highest incidence of SI individuals in the westernmost population (Pop 1) and the highest incidences of SC individuals in the easternmost populations (Pops 7 and 8; Figure 2; Table 1). The SCI within maternal lines in Pops 1–4 and 8 ranged from 0 to 1, indicating that individuals within these populations are either SI or SC (Appendix S1). By contrast, the SCI ranged from 0.28 to 1 in Pop 6 and from 0.35 to 1 in Pop 7, revealing a lack of SI individuals in our sampling of these populations (Appendix S1). Significant differences were found in SCI across populations (*F*_6, 58_ = 3.51, *P* = 0.005), and a Tukey post hoc test showed that significant differences were found only between Pop 1 and Pops 4, 7, and 8, but not among the remaining comparisons (Figure 2).

**Figure 3:**
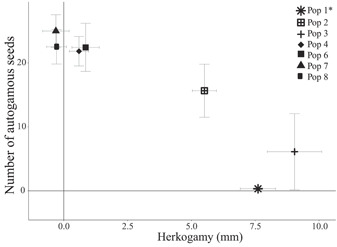
Correlation between number of seeds produced through autogamous pollination and mean herkogamy (mm) for each population of *Oenothera primiveris*, represented by different symbols. The asterisk next to Pop 1 indicates that this is the only population described as fully self‐incompatible by Wagner (2005). Error bars show SE. Pearson's correlation coefficient = −0.968, *P* = 0.0003.

Autogamy differed significantly between populations (*F*_6, 57_ = 8.6, *P* < 0.0001). The westernmost population (Pop 1) produced almost no autogamous seeds, and easternmost populations (Pops 6, 7, and 8) produced the most seeds via autogamy. In the west, Pops 2 and 3 produced intermediate numbers of autogamous seeds, while Pop 4, despite being in the west, was most similar to eastern populations (Appendix S2). A Tukey post hoc test showed that Pop 1 produced significantly fewer seeds via autogamy than Pops 2, 4, 6, 7, and 8, whereas no other comparisons were significant. Autogamous seed production was negatively correlated with herkogamy (*R* ^2^ = − 0.933, *P* = 0.002; Figure 3) and floral diameter (*R* ^2^ = − 0.82, *P* = 0.02; Appendix S4). Populations that produced few or no autogamous seeds (Pops 1, 2, and 3) had large flowers that were herkogamous, whereas those that produced the highest numbers of seeds via autogamy (Pops 4, 6, 7, and 8) had small flowers with reduced or reverse herkogamy (Appendix S2). Populations with higher SCI (Pops 4, 6, 7, and 8) had a higher number of autogamous seeds than populations with lower SCI (Pops 1, 2, and 3; *F*_1, 5_ = 82.5, *P* < 0.001), while populations with reduced flower size (Pops 4, 6, 7, and 8) produced more autogamous seeds than populations with large flower size (Pops 1, 2, and 3; *F*_1, 5_ = 10.16, *P* = 0.02).

### Floral traits

**Figure 4:**
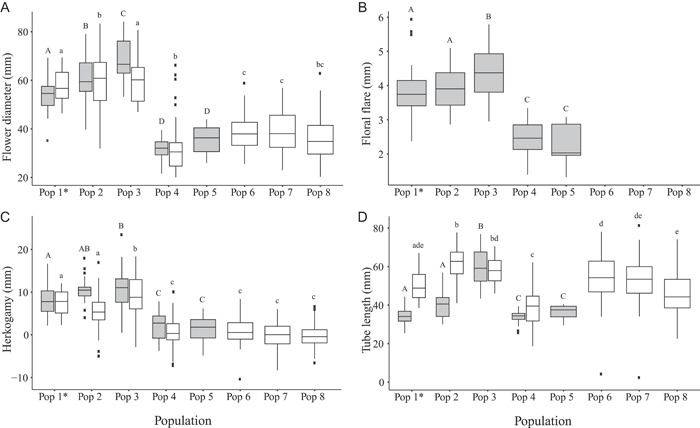
Variation in (A) floral diameter, (B) floral flare, (C) herkogamy, and (D) tube length for *Oenothera primiveris* plants evaluated in the field (gray boxes) and in the growth chamber (white boxes). Within each panel, populations sharing a letter are not significantly different from each other (Tukey post hoc test). Capital letters represent differences between populations when evaluated in the field, and lowercase letters represent differences between populations when evaluated in the growth chamber. Floral flare was only evaluated in the field (see text for further information).

**Figure 5:**
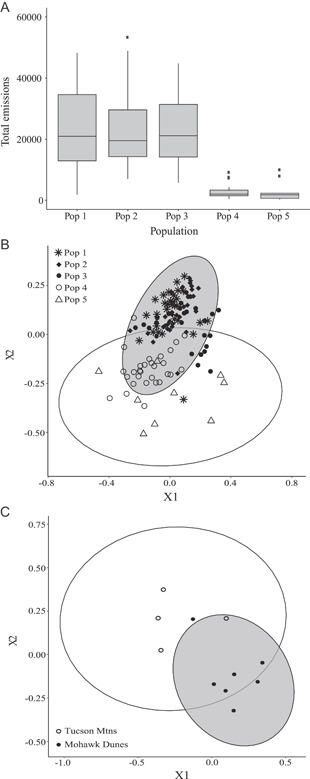
Population variation in *Oenothera primiveris* floral scent data. (A) Total emissions of scent produced (toluene equivalents/g of dry floral mass/h) from wild populations sampled in 2015 and 2016. Bars that share a letter are not significantly different according to a Tukey post hoc test. (B) NMDS of floral scent compounds collected from wild populations sampled in 2015 and 2016. (C) NMDS of floral scent compounds collected from wild populations sampled in 2001 in Arizona. Symbols represent sampled populations, and ellipses indicate geographic region (west = gray, white = east).

Populations of *O. primiveris* sampled in natural conditions showed a bimodal distribution in floral traits and overall scent emission. Flowers from three of the four western populations (Pops 1, 2, and 3) had nearly twice as large floral diameters (mean = 61.63 mm, SE = 1.25; Figure 4A) and floral flares (mean = 4.02 mm, SE = 0.08; Figure 4B), a five‐fold increase in herkogamy (mean = 9.8 mm, SE = 0.42; Figure 4C), longer floral tubes (mean = 44.4 mm, SE = 1.36; Figure 4D), and almost a ten‐fold difference in scent emission rates (mean = 25.2 µg per flower, SE = 2.79; Table 1) compared with one of the western populations (Pop 4) and the eastern population (Pop 5). All differences in floral traits between the three populations in the west (Pops 1, 2, and 3) and Pops 4 (west) and 5 (east) were significant (flower diameter: *F*_4, 121_ = 107.8, *P* < 0.0001; herkogamy: *F*_4, 114_ = 30.24, *P* < 0.0001; floral flare: *F*_4, 121_ = 40.8, *P* < 0.0001; floral tube: *F*_4, 114_ = 77.16, *P* < 0.0001; floral scent emission rate: *F*_4, 121_ = 27.08, *P* < 0.0001; Figure 5A).

Three floral traits also were measured in the growth chamber on plants grown from seven of the eight populations: four in the west (Pops 1, 2, 3, and 4) and three in the east (Pops 6, 7, and 8). Three of the western populations (Pops 1, 2, and 3) had flowers that were on average larger, with a nearly two‐fold difference in flower diameters (mean = 59.03 mm, SE = 1.28; Figure 4A), a 30‐fold increase in herkogamy (mean = 6.5 mm, SE = 0.47; Figure 4C), and 1.3× longer floral tubes (mean = 60.53 mm, SE = 1.39; Figure 4D) compared with Pop 4 (west) and all four eastern populations. Differences in floral traits between three of the populations in the west (Pops 1, 2, and 3) and Pops 4, 6, 7, and 8 were significant (flower diameter: *F*_6, 57_ = 30.68, *P* < 0.0001 [Figure 4A]; herkogamy: *F*_6, 57_ = 16.36, *P* < 0.0001 [Figure 4C]; and floral tube length: *F*_6, 57_ = 16.13, *P* < 0.0001 [Figure 4D]).

Flowers of *O. primiveris* growing in the field in 2015 and 2016 emitted 37 volatile organic compounds (VOCs), including aliphatic, aromatic, monoterpene, sesquiterpene, and nitrogenous volatiles derived from amino acids (Appendix S5). Mean floral scent emission rates were higher in three of the four western populations (Pops 1, 2, and 3; Figure 5A) when calculated per flower (7.2‐fold), and these differences persisted when standardized per gram dry mass (2.4‐fold; Appendix S5). Chemical composition of floral scent also differed between western and eastern populations (ANOSIM *R* = 0.76, *P* = 0.001), with Pops 1, 2, and 3 emitting more compounds (16.8 VOCs), on average, than those of Pop 4 (west) and Pop 5 (east) (7.2 VOCs; Figure 5B). Although scent composition range‐wide was dominated by trans‐β‐ocimene, nitrogenous aldoximes (3‐methylbutyl aldoxime, 2‐methylbutyl aldoxime), and sesquiterpenes (α‐farnesene isomers and trans‐β‐caryophyllene), floral scent from Pops 4 and 5 lacked aromatic compounds (methyl benzoate, benzyl benzoate) and long‐chain fatty acid methyl esters (methyl palmitate, methyl linoleate) characteristic of scent from three of the western populations (Appendix S5). Finally, plants from Pop 3 consistently emitted nine structurally related monoterpene volatiles, dominated by sabinene and 1,8‐cineole, that were not found in any other population of *O. primiveris* (Appendix S5).

Floral scent collected from greenhouse‐grown plants at the University of Arizona showed similar patterns to those from wild plants, with 38 identified VOCs belonging to the same biosynthetic classes, again dominated by (*E*)‐β‐ocimene, (*E,E*)‐α‐farnesene, and 3‐methylbutyl aldoxime (Appendix S6). Consistent with field data, the total scent emission was greater in plants grown from a western population (Mohawk Dunes) than those grown from an eastern population (Tucson Mountains), whether calculated per flower (14.5‐fold), per gram fresh mass (7.3‐fold), or per gram dry mass (7.9‐fold; Appendix S6). Similarly, the chemical composition also differed between Mohawk Dunes (west) and Tucson Mountains (east) (ANOSIM *R* = 0.70, *P* < 0.01; Figure 5C), with plants from Tucson Mountains producing four novel aromatic compounds and having a higher proportion of (*E,E*)‐α‐farnesene in their total emissions (Appendix S6).

### Next‐generation sequencing and genetic parameters

An average of 1,365,768 reads per individual were generated from paired‐end Illumina HiSeq runs, of which an average of 688,966 reads per individual were aligned into putative loci using the USTACKS program. After filtering for loci present in 70% of individuals per population and found in at least four populations, the average number of loci retained in each population ranged from 181,467 to 216,359. Using these strict filtering parameters, a total of 601 SNPs (~0.28%) were variable and found across all populations and were used to generate the data sets for further genetic analysis. A comparison of percentage polymorphic SNPs (%P) identified in each population found a large split across the range, with three populations in the west (Pops 1, 2, and 3) having two‐ to three‐fold more polymorphic SNPs (ranging from 46% to 60%) than the remaining west population and the two sampled populations in the east (Pops 4, 6, and 7; ranging from 17% to 21%). This pattern was replicated for all genetic diversity parameters (*N*, *N*_A_, *H*_E_), where there was a decline from west to east, with lowest diversity in most eastern populations (Table 2). This was also reflected in measures of effective population sizes, with one population (Pop 6) in the east estimated to have fewer than six founders.

Patterns of variation in many genetic parameters were correlated with population mean flower size, herkogamy, and SCI. Populations with large flowers and higher herkogamy (Pops 1, 2, and 3) had, on average, higher genetic diversity measured as %P, N, *N*_A_, and *H*_E_ than populations with small flowers (Table 2). Genetic diversity estimators %P, N, *N*_A_, and *H*_E_ showed a significant positive correlation with flower size (%P: *R* ^2^ = 0.91, *P* = 0.01; N: *R* ^2^ = 0.9, *P* = 0.01; *N*_A_: *R* ^2^ = 0.88, *P* = 0.02; *H*_E_: *R* ^2^ = 0.92, *P* = 0.01), and with herkogamy (%P: *R* ^2^ = 0.96, *P* = 0.002; N: *R* ^2^ = 0.96, *P* = 0.002; *N*_A_: *R* ^2^ = 0.95, *P* = 0.003; *H*_E_: *R* ^2^ = 0.97, *P* = 0.0015). Similarly, %P, N, N_A_, and *H*_E_ showed a significant negative correlation with SCI (%P: *R* ^2^ = −0.98, *P* < 0.001; N: *R* ^2^ = −0.98, *P* = 0.0003; *N*_A_: *R* ^2^ = −0.98, *P* < 0.001; *H*_E_: *R* ^2^ = −0.98, *P* = 0.0007). Even though all populations evaluated showed low values of *N*_E_, populations with large flowers (Pops 1, 2, and 3) had higher values of *N*_E_ than populations with small flowers (Table 2).

The inbreeding coefficient (*F*_IS_) varied from −0.15 to 0.58 across the range and showed an east‐west split. The westernmost population (Pop 1) showed reduced inbreeding, and the remaining western populations had moderate inbreeding (Pops 2 and 3) or negative inbreeding (Pop 4), which may indicate assortative mating (Table 2). The two eastern populations for which genetic data were collected (Pops 6 and 7) had very high levels of inbreeding. The inbreeding coefficient was not significantly correlated with flower size (*R* ^2^ = −0.21, *P* = 0.69), nor with herkogamy (*R* ^2^ = −0.44, *P* = 0.37) or SCI value (*R* ^2^ = 0.54, *P* = 0.27). This pattern was mirrored in the estimates of selfing rate, where the four western populations showed moderate levels of selfing and low to moderate levels of inbreeding, while the two eastern population had higher selfing rates, associated with increased autogamy (Table 2).

**Figure 6:**
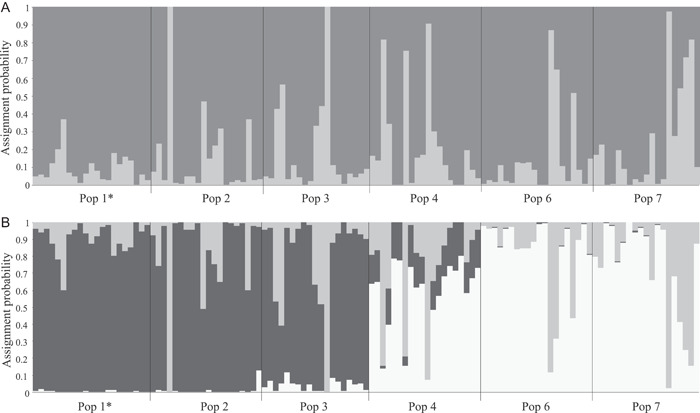
Population differentiation detected among six populations of *Oenothera primiveris* based on 601 SNP's. (A) ∆K = 2 represent the most likely number of conceptual populations. (B) ∆K = 3 represents the second most likely number of conceptual clusters and differentiates between west and east populations.

Genetic differentiation across the 1200 km range sampled (Appendix S7) was moderately high (mean *F*_ST_ = 0.16, SE = 0.01). The pairwise distances between populations with large flowers were lowest (mean *F*_ST_ = 0.07, SE = 0.004), compared to pairwise distances between populations with small flowers (mean *F*_ST_ = 0.14, SE = 0.04), whereas comparisons between populations with large and small flower sizes showed the greatest level of differentiation (mean *F*_ST_ = 0.18, SE = 0.01) (Table 3; Appendix S7), as supported by the Bayesian analysis in Structure (Pritchard et al., 2000), where the optimal ∆K = 3 differentiated populations with large flowers from those with small flowers (Figure 6).

**Table 3:** Matrix of pairwise comparison of genetic distance (*F*_ST_; below the diagonal) and spatial distance (km; above the diagonal) between populations of *Oenothera primiveris.*

	Pop 1	Pop 2	Pop 3	Pop 4	Pop 6	Pop 7
**Pop 1**	*	287.7	409.0	448.3	1182.4	994.7
**Pop 2**	0.07	*	241.0	171.5	903.0	711.0
**Pop 3**	0.06	0.08	*	194.8	823.0	658.6
**Pop 4**	0.16	0.17	0.13	*	734.8	546.7
**Pop 6**	0.20	0.23	0.18	0.19	*	202.1
**Pop 7**	0.19	0.22	0.18	0.17	0.06	*

## DISCUSSION

Our evaluation of reproductive systems, floral traits, and genetic diversity parameters across the distribution of *O. primiveris* is consistent with the hypothesis that populations capable of self‐fertilization have evolved reduced floral traits, increased autogamy, higher inbreeding, increased selfing rate, and greater population differentiation. Our study of geographic variation in breeding system across the distribution of *O. primiveris* was consistent with the findings of Wagner (2005). The number of SI individuals in each population declined from west to east, with no SI individuals found in some eastern populations. The easternmost populations had the highest SCI values, and there was an associated reduction in floral traits, namely smaller flowers, reduced herkogamy, and lower scent emission rates. These changes were associated with an increased likelihood of autogamous self‐pollination. Although the reduced floral forms do not preclude animal pollination, the high inbreeding values and selfing rates suggest that self‐fertilization is the predominant form of reproduction in eastern populations. By contrast, three of the four western populations had low self‐compatibility indices, had larger flowers, and produced more scent, consistent with more outcrossing and a stable mixed mating state. The low genetic differentiation, low inbreeding values, and low selfing rates in large‐flowered populations suggest that outcrossing via insect pollination is common. The differences in reproductive systems, floral traits, and genetic diversity parameters indicate that *O. primiveris* has experienced different evolutionary forces and ecological contexts at the extremes of its range, which alter the cost‐benefit ratio of reproductive assurance through self‐fertilization.

Contrary to our expectation, we saw not a clinal shift in traits associated with reproductive assurance but rather an abrupt transition, producing a bimodal split in all metrics. The bimodal shift suggests that there is an abrupt transition from mixed mating to complete selfing, with intermediate states being unstable (Lande and Schemske, 1985; Rausher and Chang, 1999). This transition produced a strong genetic signature with flower shape, herkogamy, and SCI being better predictors of genetic clusters and relatedness than either geographic distance or inbreeding. There was high genetic differentiation for all population pairs, which suggests some levels of reproductive isolation and potential divergence between selfing and mixed mating populations (Lowe and Allendorf, 2010). The exception was the three large‐flowered populations which had high connectivity, having values more consistent with other hawkmoth‐pollinated species, where gene flow rates are high and population differentiation is limited (Finger et al., 2014; Skogen et al., 2019). The bimodal shift was best demonstrated by one western population, Pop 4, which was genetically most similar to small‐flowered populations but was geographically closest to the three large flowered populations. This population had some SI individuals, low inbreeding values, and low selfing rates similar to the other western populations, but also had smaller flowers, reduced herkogamy, and high SCI, characteristics more similar to eastern populations. The floral changes and increased autogamy suggest there are fitness advantages to reproductive assurance in this population, while the low selfing rate and low historic inbreeding would imply that there remain advantages to avoiding inbreeding. This conflict is somewhat reflected in the negative inbreeding values that suggest assortative mating may be happening, which is often associated with strong linkage disequilibrium due to mate limitation (Sujii et al., 2015), heterosis (Oakley et al., 2019), or selection against homozygosity associated with elevated inbreeding depression (Charlesworth and Charlesworth, 1987; Aguilar et al., 2019). The contradictory attributes of this population, as well as geographic proximity to western populations, suggest that it may represent an area where the conflicting advantages associated with reproductive assurance and maintenance of outcrossing fluctuate more frequently than at the range edges.

The loss of SI is the first key step towards the evolution of selfing. Breeding system has been shown to be evolutionarily labile in Onagraceae, in some instances more so than life history (Theiss et al., 2010). While Wagner (2005) did not identify SC individuals in the Eureka Dunes population (Pop 1), our breeding system assessment revealed low levels of SC individuals in all populations. This suggests that the mating system of most populations of *O. primiveris* can vary from outcrossing to mixed mating, and some with increased selfing rates. Given that SI is the ancestral state for the family Onagraceae (Raven, 1979), the breakdown of SI in this system is possibly due to abiotic factors (Theiss et al., 2010) or the reduction of mate availability due to low S‐allele diversity (Porcher and Lande, 2005; Busch and Schoen, 2008). Wagner (2005) suggested that the shift in habitat type from sand dunes to river washes and rocky outcrops was likely the initial driver in the loss of SI in this species. Self‐compatibility and reproductive assurance can facilitate range expansion, especially for populations founded by only a few individuals (Baker, 1955; Husband and Barrett, 1991; Cheptou, 2012). The shift in SI documented range‐wide aligns with the prediction of range expansion from sand dune systems to river washes and rocky outcrops for *O. primiveris*. Dune systems are more continuous and larger in scope and therefore can support larger populations than river washes and rocky outcrops. Changes in environmental conditions and demographic factors can facilitate changes in the breeding system (Charlesworth, 2006; Igic et al., 2008; Shao et al., 2019) by presenting a release from limitation for reproductive success in obligate outcrossers.

The high effective population size and higher genetic diversity in the west are indicative of larger population size or more common ongoing gene‐flow between population facilitated by hawkmoth pollinators that travel long distances (Stockhouse, 1973; Linhart and Mendenhall, 1977) and facilitate high pollen dispersal (Finger et al., 2014; Skogen et al., 2019). Range expansion into habitats that are smaller in size (river washes and rocky outcrops), with more frequent disturbances (rain events that can wash away individuals on a regular basis), can result in large fluctuations in population sizes. Such populations are more likely to be too small to maintain sufficient S‐allele diversity for compatible mating events to occur (Busch and Schoen, 2008). This demographic change could also result in reduced pollinator services in small or fragmented populations that are more difficult for pollinators to find (Allee effect; Lamont et al., 1993; Groom, 1998), which may rapidly select for self‐compatibility. Indeed, populations of *O. primiveris* in the eastern edge of range had the highest selfing rates along with lower effective population sizes and genetic diversity, especially when compared with other, more widespread plant populations (Suárez‐Montes et al., 2016). This genetic pattern of decreasing genetic diversity and effective population size from west to east is consistent with signatures of range expansion (Hu et al., 2009) that would have been facilitated by the increasing advantage of reproductive assurance through selfing. Hence, breeding system variation may reflect an evolutionary mating strategy in the face of outcrossing limitations in colonization of new areas (Porcher and Lande, 2005; Busch and Schoen, 2008).

Once SI has been lost, the maintenance of a mixed mating system is an important dynamic factor within populations (Goodwillie et al., 2005). Across populations of *O. primiveris*, inbreeding rates varied from low to very high, with an abrupt reproductive shift from outcrossing to predominantly selfing. The mixed mating populations in the west (Pops 1 and 3) had the lowest number of SC individuals, larger flowers with higher scent emission rates, traits important to pollinator attraction, and low levels of genetic differentiation, suggestive of a selective advantage to outcrossing in these locations. By contrast, the eastern populations showed almost a complete shift to SC individuals, with smaller floral diameters, reduced or no herkogamy, and lower scent emission rates, that were capable of producing high numbers of seeds through autogamy. Although the smaller flowers of *O. primiveris* remain capable of attracting hawkmoth pollinators, as evidenced by the presence of hawkmoth scales on stigmas in small‐flowered Arizona populations (Appendix S8), the elevated inbreeding levels suggest that there is frequent selfing in these populations and that they benefit from reproductive assurance. Of the morphological changes most associated with complete selfing, the reduction in herkogamy was likely a critical step. This allowed for increased autogamous seed set (Opedal, 2018), elevating rates of self‐fertilization and levels of inbreeding, as has been seen in other species (e.g., *Ipomoea lacunosa* L., Duncan and Rausher, 2013a; *Camissoniopsis cheiranthifolia*, Dart et al., 2012; *Arabis alpine* Krock. ex Steud., Tedder et al., 2015; *Linaria cavanillesii*, Voillemot and Pannell, 2017; *Datura inoxia* Mill., Jiménez‐Lobato and Núñez‐Farfán, 2021; *Mimulus guttatus* species complex, Bodbyl Roels and Kelly, 2011).

Pollinator limitation may partially explain the observed pattern of floral trait variation and the increase in selfing rates from west to east. Populations in the west of the distribution had floral traits consistent with hawkmoth pollination (large, lightly colored, fragrant, nectariferous flowers with evening anthesis), many of which are reduced in populations in the east. Reductions in floral size, scent emission rates, and chemical complexity have been documented for other plant species that show variation in selfing rates, including moth‐pollinated species such as *O. flava* Garrett (Raguso et al., 2007) and *Abronia umbellata* Lam. (Doubleday et al., 2013). Floral traits are crucial for pollinator attraction and for promoting outcrossing; however, when pollinator visitation is unreliable or inconsistent, selective pressures on those traits can be relaxed and over time, floral traits important in pollinator attraction and fidelity can be diminished or lost (Bodbyl Roels and Kelly, 2011). In such situations, autogamy confers reproductive assurance, and selfing evolves to be the primary mode of reproduction. While hawkmoth visitation can be infrequent and is known to be variable in space and time (Miller, 1981; Campbell et al., 1997), hawkmoths are common throughout the desert southwest (Grant, 1983), are known to visit other *Oenothera* species in this geographic region (Gregory, 1964; Jogesh et al., 2016), and have been observed visiting *O. primiveris* (Appendices S3 and S8). However, while possible, the elevated inbreeding values and high genetic differentiation suggest that cross‐pollination is not frequent in the easternmost populations sampled. For these reasons, we believe that pollination by hawkmoths (and therefore outcrossing), while infrequent, is sufficient to avoid the complete evolution of selfing and extreme floral reduction (i.e., cleistogamy as in *O. flava*; Summers et al., 2015).

While demographic factors may explain the transition toward a predominantly SC breeding system and small effective population sizes, they alone do not explain the increase in autogamy observed across the distribution. Even though reproductive assurance allows for reproduction under unfavorable environmental conditions and/or in the absence of pollinators (Lloyd, 1979), in the long run, increased self‐fertilization can lead to higher inbreeding, increasing extinction risk from inbreeding depression (Frankham, 2005; Frankham et al., 2017; Cheptou, 2019). The expectation is that genetic load will be lowest in small populations, where the genetic load is often fixed (Lohr and Haag, 2015). In the short term, self‐fertilization increases the expression of the genetic load and, in large populations, can reduce the effects of inbreeding depression due to selection against deleterious alleles (purging) (Dudash and Carr, 1998). The gradual shift in the SCI in large populations along with decreasing effective population sizes and decreasing genetic diversity in small populations across the range of *O. primiveris* would provide optimal conditions for a gradual increased expression of the genetic load, which would facilitate purging to eliminate the cost of inbreeding depression (López‐Cortegano et al., 2016; Caballero et al., 2017), although this would need to be confirmed through inbreeding experiments.

## CONCLUSIONS

The pattern of loss of self‐incompatibility and reduced genetic diversity across the range of *O. primiveris* is consistent with expectations for range expansion from west to east. The abrupt transition from mixed mating to predominantly self‐fertilization is likely associated with selection for increased reproductive assurance facilitated by a reduction of floral traits. This shift would have allowed populations to establish in smaller but more variable habitat types. Even though mating system was not directly evaluated here, the increased autogamy and elevated inbreeding values of the populations in the east suggest that selfing is the predominant mating system in these populations and that outcrossing is uncommon. Populations in the west show variation in the breeding system, low genetic differentiation, low inbreeding, and floral traits important for the attraction of pollinators, suggesting that mixed mating and outcrossing are the predominant mating systems in this portion of the range. This pattern suggests that there is little advantage to reproductive assurance or that there is a high cost to selfing in western populations.

The morphological and reproductive shift across the species distribution is accompanied by a clear genetic differentiation across the range, with evidence of reproductive isolation between east and west. This divergence is associated with a shift in floral traits. These differences could conceivably continue to accumulate over evolutionary time and could eventually lead to speciation. Species that have evolved a selfing syndrome are expected to present increased autogamy, high levels of inbreeding, and low genetic diversity, and although we did see all these changes in the eastern populations, it remains to be determined if there are fitness consequences that may impact the long‐term population persistence and further evolutionary change. Overall, our data suggest that the processes driving this evolutionary shift are likely ongoing, and future work in this system should focus on identifying the relative contributions of demographic factors and pollinator limitation to the breeding system transition within and between populations. Future work should also focus on the barriers limiting gene flow between western and eastern populations that could be driving differentiation in this system. This work contributes to a better understanding of the variability of reproductive systems and traits across populations, the influence that floral traits have on reproduction, and the effects that these changes can have on the population genetic patterns.

## AUTHOR CONTRIBUTIONS

A.C.F. and J.B.F. designed the growth chamber experiment and A.C.F. conducted the experiment and analyzed the data. K.A.S. designed the field experiments and T.J. collected and analyzed the field data. T.J. and R.A.R. collected floral scent data and R.A.R. and G.T.B. processed them. A.C.F., J.B.F., and K.A.S. wrote the manuscript and all authors contributed to revisions. Funding was acquired by A.C.F., J.B.F., and K.A.S.

## Supporting information


**Appendix S1**. Self‐compatibility index (SCI) for each population and information about maternal lines evaluated.

**Appendix S2**. Number of seeds produced through autogamous pollination for each population, mean flower diameter and mean herkogamy values for the population.

**Appendix S3**. Pollinator visitation rates and assessments of hawkmoth visitation in natural populations.

**Appendix S4**. Correlation between number of seeds produced through autogamous pollination and mean diameter (mm) for each population.

**Appendix S5**. Summary of floral scent composition and emission rates from natural populations.

**Appendix S6**. Summary of floral scent composition and emission rates from common greenhouse study, 2001.

**Appendix S7**. Comparison of pairwise genetic and spatial distance among six populations.

**Appendix S8**. Visual evaluation of pollen and moth scales deposition in stigmas of natural populations during 2001 flowering season.

## Data Availability

All data and scripts used to tun the analysis and generate figures are available at Zenodo: https://doi.org/10.5281/zenodo.6342256
