# Supplementary material for: Evolution of selfing syndrome and its influence on genetic diversity and inbreeding: A range‐wide study in Oenothera primiveris
Source: Am J Bot. 2022 May 21;109(5):789–805. doi: 10.1002/ajb2.1861 (PMC9320852; doi:10.1002/ajb2.1861)
Supplement: Supplementary file 1 — Appendix S1. Self‐compatibility index (SCI) for each population and information about maternal lines evaluated. [file AJB2-109-789-s007.pdf]

Additional Information Click here to access/download;Additional Information;Appendix S1.docx

Appendix S1. **Self-compatibility index (SCI) for each population and information about maternal lines evaluated.**

Number of maternal lines used to evaluate their self-compatibility index (SCI), including number of flowers pollinated, the average number of individuals evaluated per maternal line, the average number of seed produced by crosses made in the population and standard errors reported for each evaluated trait. Average SCI across maternal lines and in parenthesis the variation observed across maternal lines.

Population	Number of maternal lines		Number of pollinated flowers		Average number of individuals evaluated per maternal line (SE)		Average number of seeds per flower crossed (SE)		Average SCI across maternal lines and their variation
	Self	*Outcross*	*Self*	*Outcross*	*Self*	*Outcross*	*Self*	*Outcross*	
Pop 1	9	5	18	8	1.44 (0.24)	1.4 (0.2)	2.7 (3)	20.6 (8.5)	0.13 (0 – 1)
Pop 2	10	11	27	21	2.1 (0.31)	1.45 (0.17)	10.6 (3.4)	16.1 (4.2)	0.39 (0 – 1)
Pop 3	5	5	6	6	1.2 (0.18)	1.2 (0.28)	23.8 (15.5)	40 (13.8)	0.29 (0 – 1)
Pop 4	11	11	37	33	2.45 (0.38)	2.18 (0.4)	24.2 (3.54)	31.6 (3.5)	0.67 (0 – 1)
Pop 6	5	5	30	8	4.4 (0.61)	1.75 (0.36)	22.1 (3.9)	31.1 (6.5)	0.69 (0.28 – 1)
Pop 7	5	5	32	21	4.6 (0.65)	3.2 (0.11)	20.2 (4.1)	25.6 (5.6)	0.81 (0.35 – 1)
Pop 8	12	11	42	31	2.67 (0.32)	2.27 (0.25)	26.2 (3.5)	21.5 (3.4)	0.77 (0 – 1)
