# Supplementary material for: Evolution of selfing syndrome and its influence on genetic diversity and inbreeding: A range‐wide study in Oenothera primiveris
Source: Am J Bot. 2022 May 21;109(5):789–805. doi: 10.1002/ajb2.1861 (PMC9320852; doi:10.1002/ajb2.1861)
Supplement: Supplementary file 2 — Appendix S2. Number of seeds produced through autogamous pollination for each population, mean flower diameter and mean herkogamy values for the population. [file AJB2-109-789-s004.pdf]

 Click here to access/download;Additional Information;Appendix S2.docx

Cisternas-Fuentes et al. – *American Journal of Botany* 2022 – Appendix S2

Appendix S2. Number seeds produced through autogamous pollination for each population, mean flower diameter and mean herkogamy values for the population.

Mean number of seeds produced through autogamy (unmanipulated flowers), flower diameter and herkogamy (measured to the nearest mm) for each population. Number of maternal lines evaluated, number of fruits or flowers measured, average number of individuals used across maternal lines, average number of flowers measured by maternal lines used and standard errors reported for each evaluated trait.

Population	Number of maternal lines evaluated	Number of fruits measured	Average number of individuals evaluated per maternal line (SE)	Average number of autogamous seeds (SE)	Number of maternal lines evaluated	Number of flowers measured	Average number of flowers measured per maternal line (SE)	Flower diameter (SE)	Herkogamy (SE)
Pop 1	7	17	1.86 (0.3)	0.32 (0.32)	8	24	3 (0.52)	57.47 (1.36)	7.60 (0.6)
Pop 2	11	42	2.36 (0.35)	15.66 (4.14)	12	51	4.25 (0.47)	59.58 (1.8)	5.51 (0.58)
Pop 3	4	13	1.25 (0.22)	6.12 (5.96)	7	10	1.42 (0.25)	59.88 (3.27)	9.04 (2.06)
Pop 4	16	241	5 (0.61)	21.84 (2.31)	13	82	6.31 (0.75)	30.85 (1.02)	0.59 (0.37)
Pop 6	6	75	5.5 (0.45)	22.44 (3.76)	5	41	8.2 (0.84)	38.47 (1.23)	0.85 (0.49)
Pop 7	5	85	8.8 (0.42)	24.99 (2.53)	5	43	8.6 (0.49)	38.84 (1.41)	-0.31 (0.51)
Pop 8	15	189	5.3 (0.57)	26.53 (2.7)	14	77	5.5 (0.54)	34.38 (1.12)	-0.28 (0.3)
