# Supplementary material for: Evolution of selfing syndrome and its influence on genetic diversity and inbreeding: A range‐wide study in Oenothera primiveris
Source: Am J Bot. 2022 May 21;109(5):789–805. doi: 10.1002/ajb2.1861 (PMC9320852; doi:10.1002/ajb2.1861)
Supplement: Supplementary file 3 — Appendix S3. Pollinator visitation rates and assessments of hawkmoth visitation in natural populations. [file AJB2-109-789-s003.pdf]

Additional Information
Click here to access/download;Additional Information;Appendix S3.docx

Appendix S3. Pollinator visitation rates and assessments of hawkmoth visitation in natural populations.

Pollinator visitation rates and assessments of hawkmoth visitation in natural populations in 2015 and 2016. Pollinator visitation rates were recorded in 4 of the 5 populations for which floral trait data were collected in the field (Pops 1 - 4) and a visitor was recorded as a potential pollinator if it contacted the petals, anthers or stigma. Pollinator observations were conducted at two time periods, for 60 minutes at dusk following floral anthesis to capture hawkmoth pollination (between 18:30 and 20:30, when hawkmoth foraging is common in other *Oenothera* species; Skogen et al. 2016) and for 30 to 60 minutes the following morning (between 8:30 and 11:00) before flower senescence to capture visitation by bees. During each observation period, between 2 and 4 human observers and/or 2 to 4 GoPro cameras (GoPro Hero, San Mateo, California, USA) simultaneously monitored flowers on 15 to 56 plants. Red LED lights were used during evening observation periods to minimize disturbance to floral visitors. Pollinators included the hawkmoth, *Hyles lineata* (Sphingidae) and small solitary bees. We recorded visits from the hawkmoth, *Hyles lineata*, to three of the four western populations (Pop 1, 2, 3). Visitation rates were highest in Pop 3 (3.25 visits per flower per hour), followed by Pop 2 (2.38 visits/flower/hour) and Pop 1 (0.277 visits/flower/hour). Small bees were recorded from Pop 2 (0.15 visits/flower/hour) and Pop 4 (0.44 visits/flower/hour).

Population	Observation period	Small bee	Medium bee	Large bee	Beefly	*Hyles lineata*
Pop 1	AM	0	0	0	0	0
Pop 1	PM	0	0	0	0	0.28
Pop 2	AM	0.15	0	0	0	0
Pop 2	PM	0	0	0	0	2.38
Pop 3	AM	0	0	0	0	0

Pop 3	PM	0	0	0	0	3.25
Pop 4	AM	0.44	0	0	0	0
Pop 4	PM	0	0	0	0	0
