# Supplementary material for: Evolution of selfing syndrome and its influence on genetic diversity and inbreeding: A range‐wide study in Oenothera primiveris
Source: Am J Bot. 2022 May 21;109(5):789–805. doi: 10.1002/ajb2.1861 (PMC9320852; doi:10.1002/ajb2.1861)

Additional Information Click here to access/download;Additional Information;Appendix S4.docx

Appendix S4. Correlation between number of seeds produced through autogamous pollination and mean diameter (mm) for each population.

Populations are represented by different symbols. Error bars show SE. Pearson's correlation coefficient = -0.82, P = 0.02.

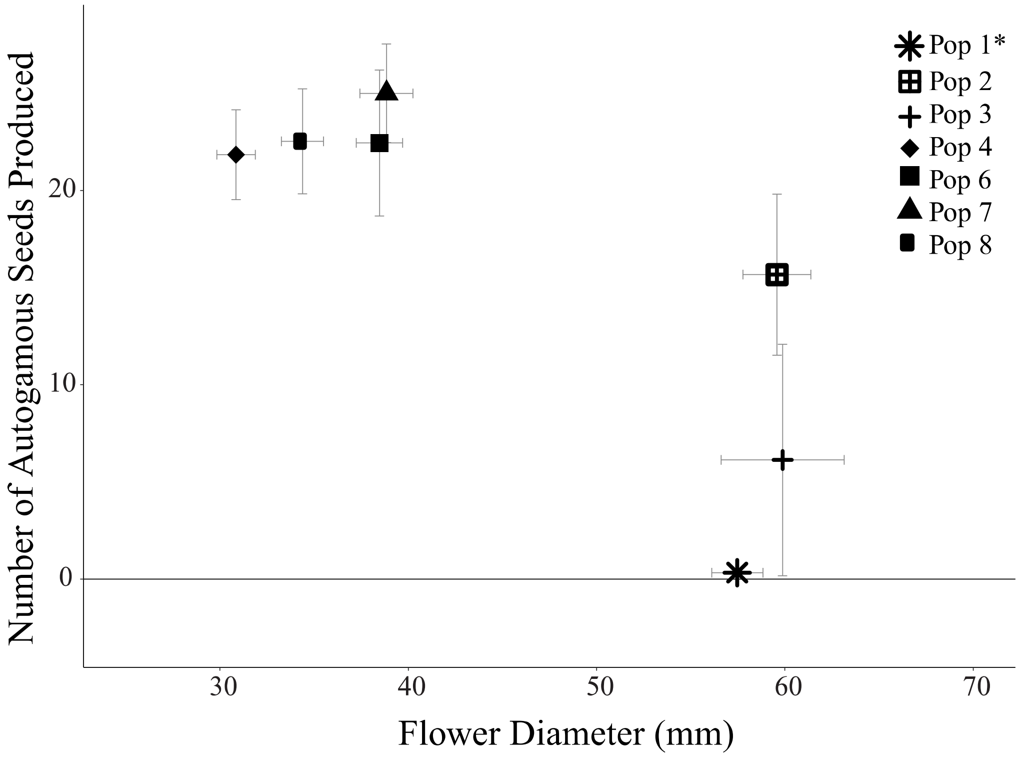

Supplement: Supplementary file 4 — Appendix S4. Correlation between number of seeds produced through autogamous pollination and mean diameter (mm) for each population. [file AJB2-109-789-s006.pdf]
