# Supplementary material for: Evolution of selfing syndrome and its influence on genetic diversity and inbreeding: A range‐wide study in Oenothera primiveris
Source: Am J Bot. 2022 May 21;109(5):789–805. doi: 10.1002/ajb2.1861 (PMC9320852; doi:10.1002/ajb2.1861)
Supplement: Supplementary file 5 — Appendix S5. Summary of floral scent composition and emission rates from natural populations. [file AJB2-109-789-s002.pdf]

Additional Information Click here to access/download;Additional Information;Appendix S5.xlsx

Appendix S5: Summary of floral scent composition and emission rates from natural populations (2015-2016)

			**Relative % of total scent emissions**									
**Scent Compounds (37)**	**Ret time**	**KI calc.**	**Pop 1: Eureka Dunes (30)**		**Pop 3: T-bone Hill (29)**		**Pop 2: Nipton Rd (30)**		**Pop 5: Whetstone Mts (9)**		**Pop 4: Hackberry Rd. (30)**	
			mean	s.e.	mean	s.e.	mean	s.e.	mean	s.e.	mean	s.e.
**Aliphatic compounds (4)**												
**(Z)-3-hexenyl acetate**	7.800	1327.3	0.03		1.46	0.90	0.51	0.10	0.31	0.08	1.02	0.11
**(Z)-3-hexen-1-ol**	8.454	1391.4	0.02	0.001	0.09	0.02	0.17	0.02	1.73	0.46		
methyl palmitate	15.071	2229.4	0.48	0.10								
methyl linoleate	16.762	2506.0	0.38	0.07	1.59	1.26	0.25	0.03				
**Monoterpenes (13)**												
tricyclene	4.336	1018.6			0.17	0.04						
**α-pinene**	4.500	1032.4			0.37	0.03						
**β-pinene**	5.481	1115.3			0.28	0.04						
**sabinene**	5.641	1129.4			2.89	1.18						
3-carene	5.950	1156.6			0.04	0.00						
**β-myrcene**	6.120	1171.6	0.16	0.01	0.15	0.01	0.16	0.01	0.15	0.00	0.12	0.01
α-terpinene	6.308	1188.1			0.04	0.02						
**1,8-cineole**	6.600	1214.6			3.86	2.34						
β-phellandrene	6.630	1217.3			0.06	0.02						
**(Z)-β-ocimene**	6.901	1242.5	0.55	0.03	0.54	0.05	0.63	0.04	0.40	0.05	0.34	0.02
**(E)-β-ocimene**	7.087	1259.7	49.37	2.88	38.96	3.80	52.29	2.87	48.38	7.81	50.34	3.03
α-terpinolene	7.449	1293.3			0.40	0.09	0.02					
**α-terpineol**	11.236	1702.1					0.62		0.19	0.02		
**Sesquiterpenes (9)**												
**(E)-β-caryophyllene**	10.541	1620.2	1.51	0.26	3.29	0.50	2.18	0.25	2.67	1.08	0.91	0.13
**β-farnesene**	10.975	1671.3	0.07	0.01	0.13	0.01			0.21	0.01		
**α-humulene**	11.161	1693.2	0.29	0.04	0.69	0.08	0.38	0.04	0.34	0.04	0.26	0.03

**(Z,E)-α-farnesene**	11.454	1729.2	0.26	0.09	1.01	0.11	0.09	0.01	1.29	0.08	0.12	
**germacrene D**	11.470	1731.1	0.02	0.003	0.03	0.01	0.03	0.00				
**(E,E)-α-farnesene**	11.653	1753.8	10.89	2.01	28.22	3.15	6.05	0.77	32.36	5.36	7.07	0.79
**caryophyllene oxide**	13.620	2013.9	7.15	1.24	4.04	0.60	6.16	0.56	17.69	6.24	4.67	0.43
**(E)-nerolidol**	13.750	2032.4	0.12						0.52	0.17	2.63	0.11
humulene oxide	13.959	2062.3	0.22	0.06	0.12	0.02	0.16	0.02	0.50	0.21		
**Aromatic compounds (5)**												
**methyl benzoate**	10.660	1634.2	0.06	0.01	0.06	0.02	0.09	0.01				
butyl benzoate	12.611	1877.3	0.03	0.01	0.02	0.00	0.07	0.02				
**3-methylbutyl benzoate**	13.000	1929.3	1.31	0.15	1.66	0.18	1.12	0.11	1.31	0.37	2.06	0.14
**(Z)-3-hexenyl benzoate**	14.457	2135.5	0.08	0.02	0.03	0.00	0.05	0.01				
**benzyl benzoate**	17.730	2655.8	0.09	0.01	0.06	0.01	0.06	0.02				
**Nitrogenous compounds (6)**												
**3-methylbutyl nitrile**	5.680	1132.8	0.23	0.02	0.10	0.01	0.09	0.01	0.25	0.00	0.44	0.07
nitro-2-methylbutane	7.806	1327.9	0.08	0.01								
nitro-3-methylbutane	7.966	1343.6	0.79	0.06	2.02	0.14	1.06	0.07	1.91		3.15	0.22
2-methylpropanaldoxime	8.391	1385.2	0.03	0.003								
**2-methylbutyraldoxime**	9.410	1491.7	3.19	0.68	0.66	0.05	1.53	0.18	1.11	0.04	0.86	0.06
**3-methylbutyraldoxime**	9.458	1496.7	33.27	1.81	34.04	2.39	33.33	2.54	41.97	9.33	36.96	2.40
**Sums, Compound Class**												
Aliphatic compounds			*0.91*		*3.14*		*0.92*		*2.04*		*1.02*	
Monoterpenoids			*50.07*		*47.75*		*53.72*		*49.12*		*50.79*	
Sesquiterpenoids			*20.54*		*37.54*		*15.04*		*55.56*		*15.65*	
Aromatic compounds			*1.56*		*1.83*		*1.39*		*1.31*		*2.06*	
Nitrogenous compounds			*37.60*		*36.83*		*36.02*		*45.24*		*41.41*	
**N; total volatiles**	128; 37		30; 27		29; 32		30; 24		9; 19		30; 10	
**# floral volatiles**			17.2	0.5	17.7	0.9	15.6	0.4	7.4	0.9	7.4	0.4
**dry mass (g) / flw**			0.1043	0.0040	0.0989	0.0045	0.0890	0.0034	0.0289	0.0039	0.0306	0.0016
**scent, µg/flw/hr**			21.786	1.974	18.944	1.937	19.292	1.825	2.891	1.183	2.628	0.329
**scent, µg/dry g flw/hr**			211.077	18.845	202.587	21.008	228.332	23.251	85.380	24.311	91.482	13.256

compounds in bold are confirmed by authentic standards
compounds in regular text show strong MS library matches (>80%)
emissions are expressed in units equivalent to the internal standard (toluene)
