# Supplementary material for: Evolution of selfing syndrome and its influence on genetic diversity and inbreeding: A range‐wide study in Oenothera primiveris
Source: Am J Bot. 2022 May 21;109(5):789–805. doi: 10.1002/ajb2.1861 (PMC9320852; doi:10.1002/ajb2.1861)
Supplement: Supplementary file 6 — Appendix S6. Summary of floral scent composition and emission rates from common greenhouse study, 2001. [file AJB2-109-789-s001.pdf]

Additional Information

Click here to access/download;Additional Information;Appendix S6.xlsx

Appendix S6: Summary of floral scent composition and emission rates from common greenhouse study, 2001

		Relative % of total scent emissions			
**Scent Compounds (38)**	**Ret time, min.**	**Mohawk Dunes (7)**		**Tucson Mts. (4)**	
		**mean**	**s.e.**	**mean**	**s.e.**
**Aliphatic compounds (6)**					
**3-methyl-1-butanol**	5.433	0.323	0.078		
isoamyl isovalerate	7.208	0.021	0.007		
**(*Z*)-3-hexenyl acetate**	7.542	0.171	0.049	0.065	0.032
**(*Z*)-3-hexen-1-ol**	8.558	0.020	0.005	0.050	
**heneicosane**	17.192	0.081	0.041	0.328	0.074
methyl linoleate	20.725	0.200	0.073	1.733	0.946
**Monoterpenes (4)**					
**β-myrcene**	4.683	0.173	0.044	0.120	0.041
**(*Z*)-β-ocimene**	6.092	0.453	0.112	0.068	0.045
**(*E*)-β-ocimene**	6.542	32.330	6.430	5.438	4.145
**α-terpineol**	12.775	<0.001	<0.001	1.215	0.385
**Sesquiterpenes (9)**					
**β-caryophyllene**	11.767	6.497	3.240	2.220	0.260
**(*E*)-β-farnesene**	12.467	0.070	0.027	0.350	0.017
**α-humulene**	12.583	0.603	0.189	0.275	0.019
**germacrene D**	13.080	0.031	0.010		
(*Z,E*)-alpha-farnesene	13.230	0.229	0.088	1.113	0.371
**(*E,E*)-α-farnesene**	13.520	5.370	2.079	24.775	8.383
caryophyllene oxide1	15.975	0.184	0.047	0.523	0.100
**caryophyllene oxide2**	16.083	4.091	1.242	8.063	2.695
humulene epoxide	16.642	0.161	0.053	2.160	1.642
**Aromatic compounds (8)**					
**methyl benzoate**	11.908	0.020	0.005	0.063	0.018
**methyl salicylate**	13.808			0.017	0.006
**benzyl alcohol**	14.792			0.220	0.000
**benzyl isovalerate**	15.067			0.075	0.004
**2-phenylethanol**	15.183			0.070	0.028
**isoamyl benzoate**	15.283	1.007	0.162	1.445	0.346
**(*Z*)-3-hexenyl benzoate**	17.417	0.017	0.007	0.080	0.028
**benzyl benzoate**	21.817	0.021	0.006	1.015	0.535
**Nitrogenous compounds (11)**					
**2-methylbutyronitrile**	3.075	0.027	0.007	0.160	

**3-methylbutyronitrile**	3.775	0.590	0.213	1.253	0.695
nitro-2-methylbutane	7.567	0.043	0.011	0.050	0.010
nitro-3-methylbutane	7.808	1.474	0.203	2.340	0.636
2-methylpropanaldoxime	8.592	0.056	0.013		
2-methylpropanaldoxime	8.867	0.040	0.008		
**2-methylbutyraldoxime**	10.150	5.111	1.111	2.033	0.258
**3-methylbutyraldoxime**	10.258	19.180	1.586	20.920	2.106
**2-methylbutyraldoxime**	10.392	3.023	0.576	1.788	0.082
**3-methylbutyraldoxime**	10.750	18.237	1.768	20.905	2.370
**phenylacetonitrile**	15.417			0.040	0.009
**Sums, Compound Class**					
Aliphatic compounds		*0.473*		*2.175*	
Monoterpenoids		*32.956*		*6.840*	
Sesquiterpenoids		*17.237*		*39.478*	
Aromatic compounds		*1.066*		*2.984*	
Nitrogenous compounds		*47.781*		*49.488*	
**N, total volatiles**	11; 38	7; 33		4; 33	
**# floral volatiles**		29.4	0.5	28.3	1.2
**fresh mass (g) / flw**		0.4807	0.0674	0.2258	0.0310
**dry mass (g) / flw**		0.0485	0.0054	0.0275	0.0049
**scent, µg/flw/hr**		20.913	4.973	1.440	0.735
**scent, µg/fresh g flw/hr**		41.736	7.641	5.704	2.261
**scent, µg/dry g flw/hr**		421.901	79.355	53.313	24.818

compounds in bold are confirmed by authentic standards

compounds in regular text show strong MS library matches (>80%)

emissions are expressed in units equivalent to the internal standard (toluene)
