# Supplementary material for: Evolution of selfing syndrome and its influence on genetic diversity and inbreeding: A range‐wide study in Oenothera primiveris
Source: Am J Bot. 2022 May 21;109(5):789–805. doi: 10.1002/ajb2.1861 (PMC9320852; doi:10.1002/ajb2.1861)

Additional Information

Click here to access/download;Additional Information;Appendix S7.docx

Appendix S7. Comparison of pairwise genetic and spatial distance among 6 populations of *O. primiveris.*

Comparison of pairwise genetic (F_{ST}/(1-F_{ST}) and geographic distance (ln(Km)) among 6 populations of *Oenothera primiveris.* Squares represent pairwise comparisons between two populations with large flowers, triangles represent pairwise comparisons between two populations with small flowers, and circles pairwise comparison between a large flowered and a small flowered population.

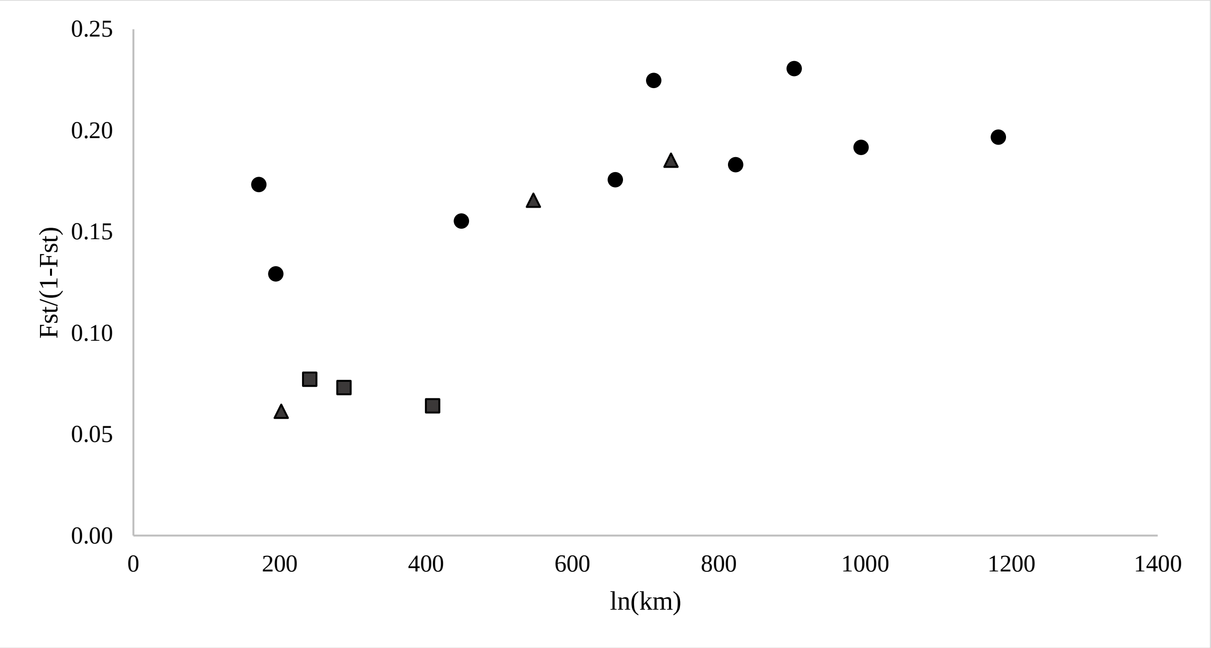

Supplement: Supplementary file 7 — Appendix S7. Comparison of pairwise genetic and spatial distance among six populations. [file AJB2-109-789-s005.pdf]
