# Supplementary material for: Evolution of selfing syndrome and its influence on genetic diversity and inbreeding: A range‐wide study in Oenothera primiveris
Source: Am J Bot. 2022 May 21;109(5):789–805. doi: 10.1002/ajb2.1861 (PMC9320852; doi:10.1002/ajb2.1861)
Supplement: Supplementary file 8 — Appendix S8. Visual evaluation of pollen and moth scales deposition in stigmas of natural populations during 2001 flowering season. [file AJB2-109-789-s008.pdf]

Additional Information Click here to access/download;Additional Information;Appendix S8.docx

Appendix S8. Visual evaluation of pollen and moth scales deposition in stigmas of natural populations during 2001 flowering season.

Methods: Considering that pollinator presence across populations can vary across space and time, populations of *O. primiveris* were surveyed opportunistically for pollen and moth scales across this species' distribution in southern Arizona during spring 2001. Populations in Arizona have reduced flower size compared to populations in the west, but variation in size within the state could lead to differences in visitation rates. In this case, the prediction was that within small-flowered populations of *O. primiveris*, hawkmoths might be more likely to visit and pollinate larger flowers with deeper nectar tubes. Transects were walked through natural populations at dawn, using a hand lens (10X) to score the presence of pollen and scales on stigmas of each open flower that had opened the previous evening. Stigmatic surfaces of *Oenothera* spp. are sticky and tend to accumulate scales after floral visits by moths, providing evidence for moth visitation in the absence of direct observation (Cruden et al., 1976; Raguso et al., 2003; Schlumpberger et al., 2009). Additional samples of flowers were collected individually into glassine envelopes and returned to the laboratory to verify pollen and scales on stigmatic surfaces using a dissecting microscope (100X magnification), to be compared with style length as a proxy for floral depth.

Results: Four of the five populations surveyed in 2001 were of small-flowered *O. primiveris* plants, nearly always producing only one flower per rosette each evening in small populations. The following patterns emerge from the data collected for these populations. Stigma deposition of moth scales was scored in the populations near Tucson but not in those near Sonoita (Organ Pipe

National Monument), and they were never observed without pollen deposition on the upper stigma surface, where it was likely to represent outcrossed rather than self-pollen (moths come into contact with stigmas when foraging for nectar). Conversely, pollen was observed on several stigmas lacking moth scales, which might be due to visitation by bees or to wind-aided autogamy. In the Tucson Mts. population, moth scales and pollen were more likely to co-occur on stigmas when styles were longer than 50 mm and it is possible that contact between moth and stigma is more likely for longer styles. These observations support the hypothesis that small-flowered *O. primiveris* plants from the central portion of the species distribution show a mixed mating system in which occasional pollination augments autogamy. A similar pattern emerged from a transect through a large-flowered population (Mohawk Dunes, AZ), in which 14 of 50 flowers surveyed had stigmatic moth scales and pollen deposition, 32 flowers had stigmatic pollen without scales, and only 4 flowers appeared to have been unvisited. However, in this population there was no obvious relationship between style length, moth scale and pollen deposition, largely due to the low frequency of sampled flowers bearing moth scales.

Population	Population ID	GPS coordinates		Flower Size	Number of flowers with scales (S+) and pollen (P+) on the stigma				Sampling	Date
		Latitude	Longitude		S+, (--)	S+, P+	(--), P+	(--), (--)	*1 flw / plt*	
Mohawk Dunes, Yuma Co., AZ	MD	32.7014597	-113.8221144	large	0	14	32	4	50	15-Mar-01
Quitobaquito Springs, Organ Pipe NM, AZ	QS	31.9445081	-113.0264199	small	0	0	3	2	5	10-Mar-01

Alamo Canyon Trailhead, Organ Pipe, AZ	AC	32.0716535	-112.7303522	small	0	0	4	0	4	11-Mar-01
Desert Peak, Pinal Co., AZ	DP	32.5864155	-111.2834311	small	0	11	5	0	16	12-Mar-01
Tucson Mts., Pima Co., AZ	TM	32.1991876	-111.061546	small	0	1	11	6	18	8-Mar-01
		32.1991876	-111.061546	small	0	0	7	11	18	13-Mar-01

References

Cruden, R. W., S. Kinsman, R. E. S. Ii, and Y. B. Linhart. 1976. Pollination, Fecundity, and the Distribution of Moth-Flowered Plants. *Biotropica* 8: 204.

Raguso, R. A., C. Henzel, S. L. Buchmann, and G. P. Nabhan. 2003. Trumpet Flowers of the Sonoran Desert: Floral Biology of *Peniocereus* Cacti and Sacred *Datura*. *International Journal of Plant Sciences* 164: 877–892.

Schlumpberger, B. O., A. A. Cocucci, M. Moré, A. N. Sérsic, and R. A. Raguso. 2009. Extreme variation in floral characters and its consequences for pollinator attraction among populations of an Andean cactus. *Annals of Botany* 103: 1489–1500.
